# MRI-based habitat radiomics analysis for predicting clinically significant prostate cancer: a retrospective dual-center study

**DOI:** 10.3389/fonc.2026.1831344

**Published:** 2026-09-21

**Authors:** Zefei Chen, Yinquan Ye, Yun Peng, Zijian Gong, Zhixuan Liu, Xiaochang Xiang, Yanliang Liao, Lianggeng Gong

**Affiliations:** 1 Department of Radiology, The Second Affiliated Hospital, Jiangxi Medical College, Nanchang University, Nanchang, China; 2 Intelligent Medical Imaging of Jiangxi Key Laboratory, Nanchang, China; 3 Department of Radiology, The First Hospital of Xiushui, Jiujiang, China; 4 Department of Radiology, Pingxiang People’s Hospital, Pingxiang, China

**Keywords:** clinically significant prostate cancer, habitat analysis, magnetic resonance imaging, PI-RADS 2.1, radiomics

## Abstract

**Objective:**

This study aims to evaluate the performance of habitat radiomics based on multi-parameter magnetic resonance (mpMRI) in predicting clinically significant prostate cancer (csPCa).

**Materials and methods:**

This retrospective study included 642 patients with pathology-proven prostate cancer (PCa) and benign prostatic hyperplasia (BPH) from two institutions. Center 1 was split into a training and internal test set, while Center 2 served as the external test set. Tumor regions of interest (ROI) were divided into three spatial habitats by K-means clustering. The classical radiomics score (Crad-score) and habitat radiomics score (Hrad-score) were developed following feature selection and dimensionality reduction. Multivariable analysis was conducted to ascertain independent predictors. The performance of these models was evaluated using receiver operating characteristic (ROC) curves. The Delong test was used to compare the area under the curves(AUCs) between models. The best-performing radiomics model was integrated with clinical data to develop a combined model, presented as a nomogram. Moreover, we further investigated whether the combined model provided additional value for improving the predictive accuracy of csPCa detection in PI-RADS 3–4 lesions.

**Results:**

The Hrad-score showed better performance than the Crad-score, achieving AUCs of 0.931, 0.907, and 0.893 in the training, internal test, and external test sets, respectively. The CH Combined model yielded optimal performance in all three sets, with AUCs of 0.966, 0.954, and 0.954, respectively. Additionally, in the subgroup of patients with PI-RADS 3–4 lesions, the CH combined model outperformed other single models in predicting csPCa, with AUCs of 0.933, 0.869 and 0.962 across the three sets.

**Conclusion:**

Habitat analysis could act as a non-invasive auxiliary tool for predicting csPCa, and furthermore, it could reduce unnecessary biopsies and assist physicians in making clinical decisions.

## 1 Introduction

Prostate cancer (PCa) is the world’s second most common cancer and the fifth leading cause of cancer-related death among men. In 2022, there were approximately 1.467 million new cases of PCa and 397,000 associated deaths, resulting in significant societal and economic burdens (1). According to the International Society of Urological Pathology (ISUP) Grade Grouping, clinically significant prostate cancer (csPCa) is defined as: Gleason score (GS)  ≥  7 (2), which is highly invasive and rapidly progressing, requiring timely diagnosis and early interventions. In contrast, the clinically insignificant PCa (ciPCa, GS < 7) and benign prostatic hyperplasia (BPH) are typically low-risk, indolent lesions that can be managed through dynamic follow-up rather than surgery (3).

Prostate biopsy remains the gold standard for diagnosing PCa, but it is an invasive procedure associated with several potential complications, including bleeding, infection, pain, etc. (4). Magnetic resonance imaging (MRI) and its derived Prostate Imaging-Reporting and Data System (PI-RADS) are currently considered the preferred non-invasive imaging method for preoperative detection of csPCa (5–8). However, as a semi-quantitative assessment tool, PI-RADS not only requires significant expertise from interpreters but also leads to inter-reader discrepancies. Studies have shown that up to 60% of patients with positive multi-parameter MRI (mpMRI) (PI-RADS ≥ 3) may still have a negative biopsy result (9), which means they will undergo unnecessary prostate biopsies and experience adverse effects.

Habitat radiomics is an emerging technique for clustering the voxels with similar characteristics within a tumor lesion and dividing them into sub-regions with specific biological properties (10, 11). Indeed, predictive models based on habitat or subregion radiomics have performed exceptionally well in various malignancies (12–14). Beig et al. proposed an MRI-derived habitat radiomics-based risk score to predict glioblastoma progression-free survival, demonstrating that tumor habitat features correlate strongly with invasive biology (15).

This study aims to explore the feasibility of mpMRI-based habitat analysis in identifying the csPCa. A combined model that integrated habitat radiomics and clinical factors was developed and validated for csPCa prediction across two centers. Moreover, in PIRADS 3–4 cases, we investigated the potential of the combined model to accurately predict csPCa.

## 2 Methods

The study protocol was approved by the Medical Research Ethics Committee of the Second Affiliated Hospital of Nanchang University (approval number: IIT-O-2024-279), and the informed consent was waived. The study complies with the requirements of the Declaration of Helsinki.

### 2.1 Patients

A total of 1671 patients with pathologically confirmed PCa or BPH were enrolled from The Second Affiliated Hospital of Nanchang University (Center 1) between January 2019 and June 2023, and from Xiushui County People’s Hospital (Center 2) between September 2020 and July 2024. All subjects underwent a definitive pathological evaluation, either through a 12-core systematic biopsy, an ultrasound fusion targeted biopsy combined with a systematic biopsy, or radical prostatectomy within one month of the initial MRI scan. The exclusion criteria were as follows: (1) incomplete clinical and imaging data; (2)history of prostate-related treatment, including endocrine therapy, radiotherapy, chemotherapy, or surgery before MRI examination; (3) incomplete or poor-quality MRI image sequences; (4) lesion size < 3 consecutive axial slices. Patients in Center 1 were randomly divided into a training set and an internal test set at a ratio of 8:2. The external test set consisted of patients from Center 2.

All feature selection procedures, including statistical tests and LASSO-based optimization, were performed solely on training data to ensure that information from the test set remained sequestered during model development.

### 2.2 Clinicopathological variables

Prostate-cancer-specific clinicopathological data, encompassing histological subtypes and Gleason grades, were ascertained from a spectrum of surgical specimens. These included specimens obtained via systematic biopsy, total prostatectomy, and palliative transurethral resection (TURP). PCa patients with a Gleason score ≥7 were categorized as the csPCa (16), whereas BPH and ciPCa with a Gleason score < 7 were categorized as the non-csPCa. The following clinical data were collected for all patients: Age, total prostate-specific antigen (PSA), prostate volume (PV), and prostate-specific antigen density (PSAD).

### 2.3 MRI acquisition and interpretation

All patients underwent mpMRI scanning, including T1WI, T2WI, and DWI (with a b-value of 3000) sequences. The images in Center 1 were acquired using three machines (GE Signa HDxt 1.5T MR, GE Signa HDxt 3.0T MR, GE Discovery 3.0T MR). The imaging data from Center 2 was obtained at 3.0T MR (MAGNETOM Skyra 3.0T MR). The specific parameters of MRI in the two centers are demonstrated in **Supplementary Table S1** (Center 1) and **Supplementary Table S2** (Center 2).

According to the PI-RADS v2.1 scoring standard, a senior radiologist with 12 years of MRI expertise who was blind to the pathological data assigned a five-point score to each patient’s prostate index lesions on mpMRI images. When multiple lesions were present, the lesion with the highest PI-RADS score on the images was defined as the index lesion.

### 2.4 Image preprocessing and ROI delineation

For MRI image preprocessing, three steps were applied to ensure comparability: normalization, resampling to a consistent voxel spacing of 1 mm × 1 mm × 1 mm, and unification of signal gray values within the range of 0–4,300.

Two radiologists (readers A and B) performed ROI segmentation in a blinded manner to assess interobserver reliability. The ROI for each patient was manually delineated by a trained radiologist (Reader A). The second radiologist (Reader B) randomly selected 50% of cases from Center 1 to perform a secondary ROIs delineation.

### 2.5 Habitat subregion division and feature extraction

The K-means algorithm was employed for initialization with a fixed random seed. To ensure that the identified habitats were biologically consistent across individuals, clustering was conducted on a pooled voxel set from the entire training cohort, deriving universal centroids that were subsequently applied to all patients. To identify the optimal number of clusters, the cluster analysis was performed, with the number of clusters ranging from 2 to 10. The Calinski-Harabasz (CH) score was used to determine the optimal number of clusters. To preserve as much microenvironmental information as possible within each habitat, missing features were imputed using the K-nearest neighbor algorithm.

All features were extracted using the pyradiomics package in Python. A total of 107 features were extracted for each lesion or subregion on each sequence, comprising 18 first-order features, 14 shape features, and 75 texture features. Texture features are extracted using the following methods: Gray-level distance-zone matrix, gray-level co-occurrence matrix, gray-level run-length matrix, gray-level size-zone matrix, and neighborhood gray tone difference matrix. The steps described above were repeated for all three sequences.

### 2.6 Feature dimension reduction

All data were standardized using Z-score normalization. Dimensionality reduction proceeded as follows: First, intraclass correlation coefficient (ICC) analysis was performed to assess the inter-rater reliability of features between readers A and B, and the features with ICC < 0.7 were excluded. Second, Pearson’s correlation analysis was used to assess the correlation between features, and only one of the two features with a correlation degree greater than 0.7 was retained. Finally, Least absolute shrinkage and selection operator(LASSO) regression based on 10-fold cross-validation for further feature selection utilizes Mean Squared Error (MSE) and the minimum lambda as the criteria for choosing the optimal features. A radiomic score (rad-score) was calculated as the linear combination of the selected radiomic features and their corresponding coefficients. Then, the whole tumor classical rad-score (Crad-score) and the habitat rad-score (Hrad-score) were calculated for each patient.

### 2.7 Statistical analysis and combined model construction

All statistical analysis were performed using Python 3.9.13 (Python Software Foundation, Wilmington, DE, USA), RStudio software (R v 4.3.2, https://www.r-project.org/), and IBM SPSS Statistics v 27. In this study, P< 0.05 was considered statistically significant. The interquartile range method was used to identify outliers, which were then replaced with the mean. The Kolmogorov–Smirnov test was used to assess the normality of the data distribution. The Mann-Whitney U test or Student’s t-test for continuous variables and the chi-square test or Fisher’s exact test for categorical variables were used to assess differences between groups. Forward stepwise selection of logistic regression models was used to select for clinically independent predictors. Multivariable logistic regression analysis was utilized to construct the combined model nomogram, which integrated the Rad-score and independent clinical factors.

The diagnostic efficacy of the model was comprehensively evaluated based on five indicators: area under the curve (AUC), sensitivity, specificity, accuracy, and F1 value. The Delong test is used for the comparison of AUC between models. Decision curve analysis (DCA) was conducted to quantify the clinical net benefit. The nomogram visualized the combined model integrating Rad-score and clinical risk factors into an easy-to-use individualized prediction of csPCa.

## 3 Result

### 3.1 Patient information

**Figure 1** shows the flow of patient selection. Finally, a total of 642 patients(mean age: 71.24 ± 7.62) were enrolled from two centers, with 554 cases from Center 1 and 88 cases from Center 2. Patients from Center 1 were randomly assigned to 443 cases in the training set (including 264 csPCa, 179 non-csPCa) and 111 cases in the internal test set (including 64 csPCa, 47 non-csPCa) at a ratio of 8:2. Patients from Center 2 were designated as the external test set (including 28 csPCa, 60 non-csPCa). There were no significant differences in age, PSA, PV, PSAD, and PI-RADS between the training and internal test sets (P>0.05). Statistical significance was observed in terms of pathology results, PSA, PSAD, and PI-RADS score (P < 0.001) between training and external test sets. Baseline characteristics of the patients are presented in **Table 1**.

**Figure 1:**
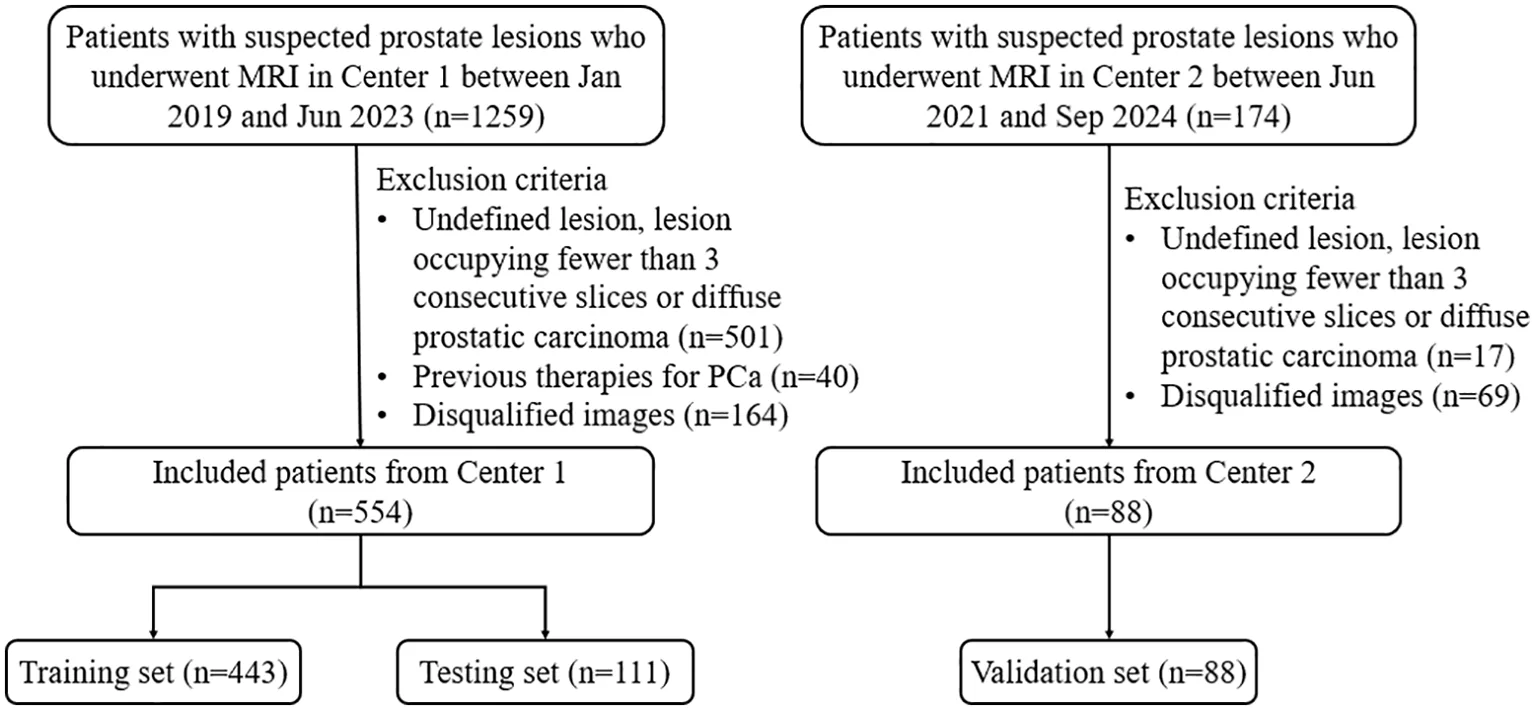
Flowchart of the inclusion and exclusion criteria.

**Table 1:** The characteristics of all patients.

	Training set (n = 443)	Test set (n = 111)	Validation set (n = 88)	P^a^	P^b^
Pathology				0.711	<0.001
cs PCa	264(59.6%)	64(57.7%)	28(31.8%)		
non-csPCa	179(40.4%)	47(42.3%)	60(68.2%)		
Age	71.1 ± 7.80	71.32 ± 7.48	71.05 ± 8.16	0.780	0.757
PSA(ng/ml)	20.10(9.81–26.40)	15.9(9.00–24.80)	8.45(3.70–14.60)	0.347	<0.001*
PV(cm^3^)	42.93(31.00–62.60)	42.01(27.58–60.54)	39.01(26.72–66.70)	0.561	0.161
PSAD	0.41(0.19–0.77)	0.41(0.20–0.74)	0.15(0.08–0.43)	0.688	<0.001*
PI–RADS				0.426	<0.001*
1	8(1.8%)	3(2.7%)	0(0.0%)		
2	50(11.3%)	8(7.2%)	4(4.5%)		
3	114(25.7%)	29(26.1%)	43(48.9%)		
4	59(13.3%)	21(18.9%)	13(14.8%)		
5	212(47.9%)	50(45.0%)	28(31.8%)		

*Data are presented as numbers, mean ± standard deviation, or median (interquartile range). P^a^, training set vs internal test set. P^b^, training set vs external test set.

csPCa, clinically significant prostate cancer. non-csPCa, combining clinically insignificant prostate cancer and benign prostatic hyperplasia. PSA, prostate-specific antigen. PSAD, prostate-specific antigen density. PV, prostate gland volume.

### 3.2 Features selection and model construction

Habitat radiomics: According to the Calinski-Harabasz value, the optimal number of cluster centers was determined as three, which was subsequently employed for sub-regional segmentation of each lesion. With 321 features extracted from each habitat, the total number of features across all habitats reached 963. After Pearson’s correlation analysis, 362 radiomic features were retained. Then, 41 radiomic features with non-zero coefficients were selected by LASSO regression (**Figures 2A–C**).

**Figure 2:**
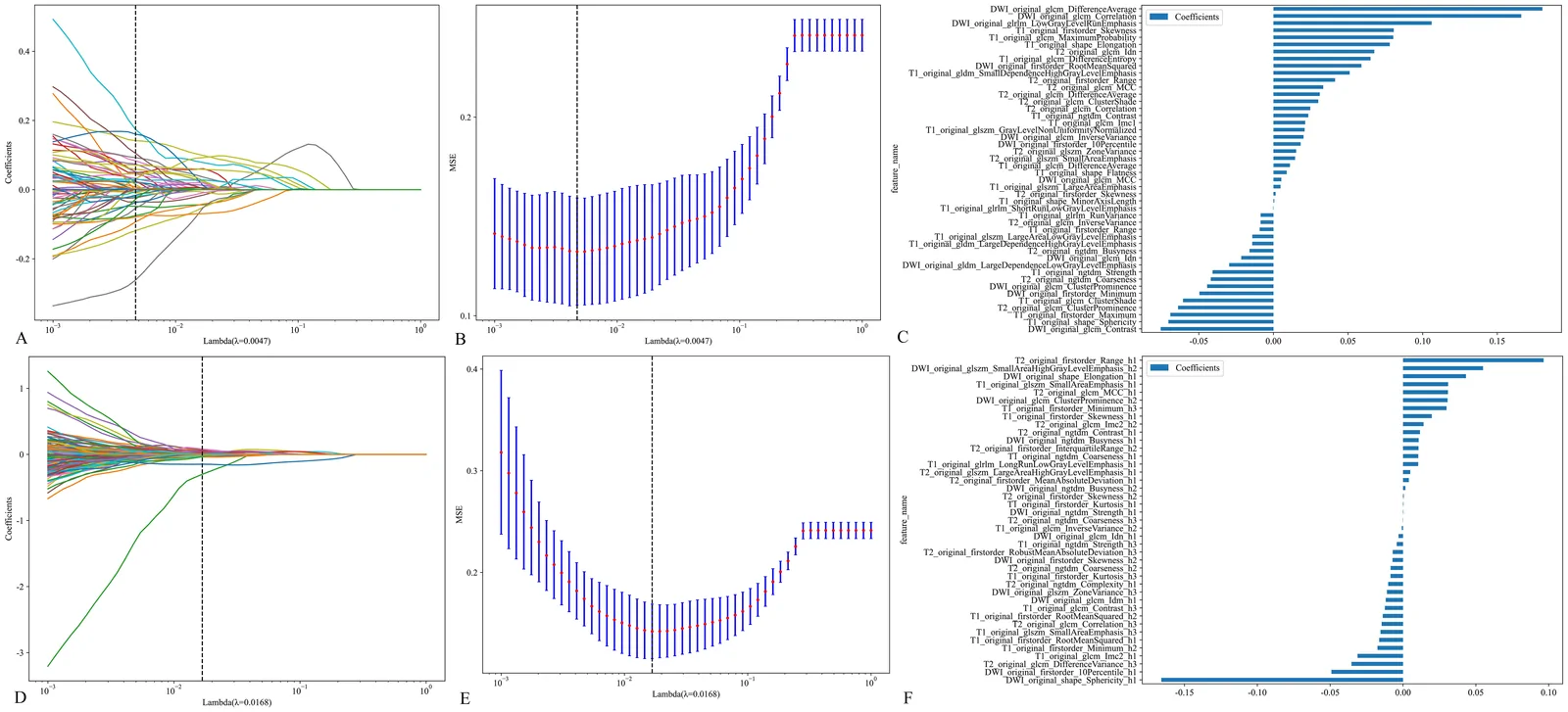
The feature screening process of the LASSO algorithm for each radiomics model. The screening process of classical radiomics features by the LASSO algorithm **(A)**, selection of λ parameters **(B)**, and the remaining features **(C)**, and the screening process of habitat radiomics features by the LASSO algorithm **(D)**, selection of λ parameters **(E)**, and the remaining features **(F)**.

Classical radiomics: A total of 321 radiomics features were obtained, with 107 features extracted from each sequence of each lesion. ICC analysis was implemented, followed by a Pearson correlation coefficient to mitigate redundancy, resulting in the acquisition of 93 features. Subsequently, the feature dimension is diminished via LASSO regression. Finally, 46 classical radiomics features were retained (**Figures 2D–F**).

Then the Hrad-score and Crad-score models were developed. The AUCs of the Crad-score for three different settings were 0.955 (95% CI:0.937-0.971), 0.888 (95% CI:0.826-0.949), and 0.857 (95% CI:0.772-0.932). The Hrad-score demonstrated AUCs of 0.931(95% CI: 0.906-0.952), 0.907(95% CI: 0.850-0.957), and 0.893 (95% CI: 0.814-0.957) in the training, internal test, and external test sets, respectively.

In the internal test and external test sets, the Hrad-score yielded higher AUCs than the Crad-score (0.907 vs 0.888, 0.893 vs 0.857; all P>0.05), but the differences were not statistically significant. Accordingly, we constructed the combined model based on the two radiomic models separately.

### 3.3 Nomogram construction and evaluation

Forward stepwise selection multivariate logistic regression identified PSAD and PI-RADS scores as independent risk factors. Based on logistic regression, the CH combined model incorporating the Hrad-score, PSAD, and PI-RADS score was constructed. Meanwhile, the CC combined model was built by integrating the Crad-score, PSAD, and PI-RADS score. The better-performing combined predictive model was visualized as a nomogram, which was used to predict the risk of csPCa (**Figure 3**).

**Figure 3:**
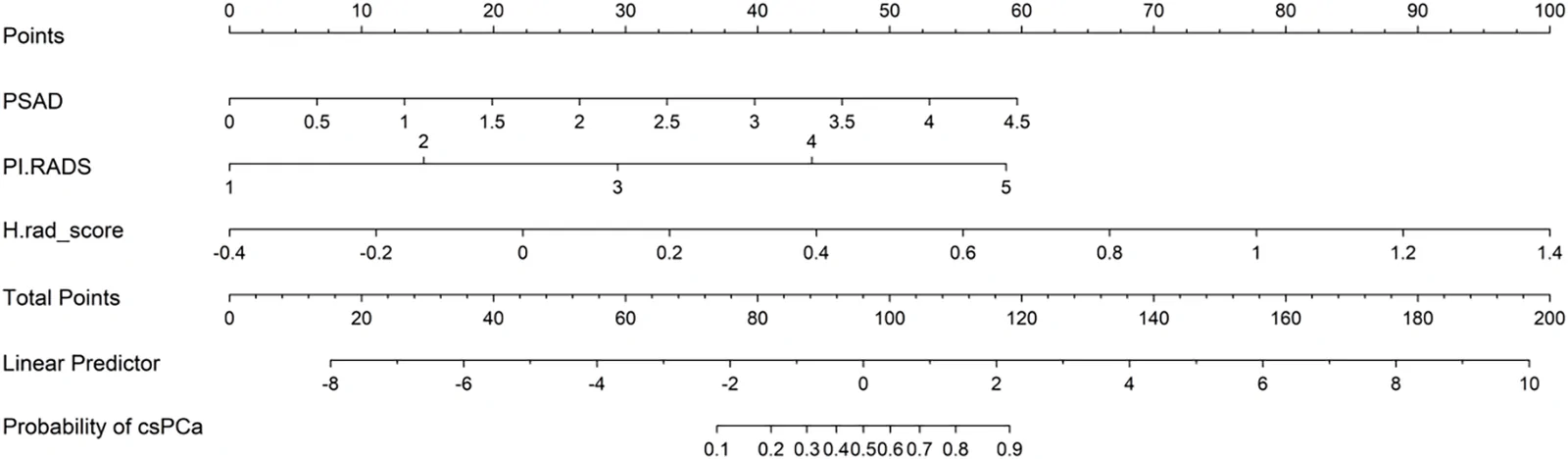
Nomogram based on a combined model constructed using clinically independent predictors and habitat radiomics score.

The results of the models are shown in **Figure 4** and **Table 2**. The CH combined model demonstrated the best performance, with AUCs of 0.966 (0.950-0.982) in the training, 0.954 (0.916-0.992) in the internal test set, and 0.954 (0.914-0.994) in the external test set. In all three sets, the CH combined model demonstrated higher performance than the clinical model (P < 0.05). The calibration curves indicated good agreement between the combined model and actual results. Decision curve analysis displayed a large net benefit of the CH combined model compared with other models.

**Figure 4:**
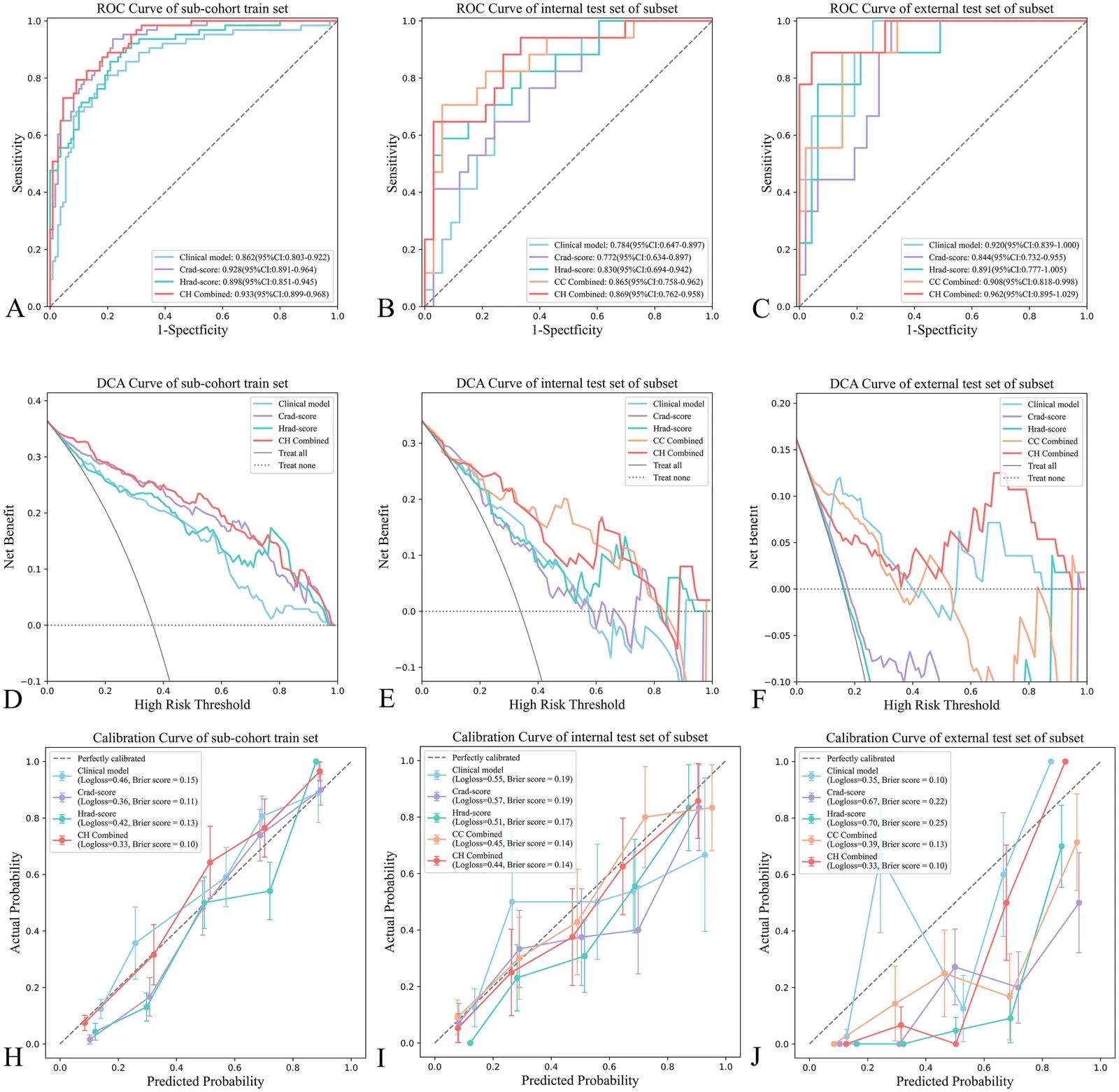
The efficacy of each model in identifying significant prostate lesions. Model evaluation; ROC curves of the different model in the train **(A)**, internal test **(B)** and external test **(C)**; the DCA curve for the train **(D)**, internal test **(E)** and external test **(F)** sets; calibration curves in the train **(G)**, internal test **(H)** and external test **(I)** sets.

**Table 2:** The predictive performance of each model in the training set, internal test set, and external test set.

Model	AUC (95% CI)	ACC (95% CI)	SEN (95% CI)	SPE (95% CI)	F1 (95% CI)	*p*
Training set						
Crad-score	0.955 (0.937-0.972)	0.833 (0.783-0.881)	0.933 (0.895-0.966)	0.948 (0.919-0.974)	0.791 (0.734-0.847)	0.200
Hrad-score	0.931 (0.909-0.954)	0.852 (0.810-0.895)	0.849 (0.799-0.903)	0.893 (0.853-0.931)	0.796 (0.735-0.853)	0.000
Clinical	0.948 (0.926-0.970)	0.920 (0.887-0.952)	0.888 (0.845-0.934)	0.924 (0.893-0.956)	0.883 (0.832-0.931)	0.002
CC Combined	0.976 (0.963-0.989)	0.962 (0.937-0.982)	0.899 (0.859-0.942)	0.934 (0.905-0.962)	0.942 (0.903-0.973)	0.500
CH Combined	0.966 (0.950-0.982)	0.932 (0.900-0.962)	0.916 (0.875-0.955)	0.943 (0.915-0.970)	0.901 (0.855-0.944)
Internal test set						
Crad-score	0.888 (0.826-0.950)	0.811 (0.739-0.883)	0.797 (0.700-0.889)	0.830 (0.720-0.933)	0.829 (0.759-0.896)	0.020
Hrad-score	0.907 (0.852-0.962)	0.829 (0.757-0.901)	0.891 (0.812-0.962)	0.745 (0.615-0.872)	0.857 (0.792-0.919)	0.024
Clinical	0.931 (0.884-0.978)	0.847 (0.784-0.910)	0.906 (0.831-0.967)	0.766 (0.646-0.886)	0.872 (0.810-0.929)	0.044
CC Combined	0.947 (0.906-0.987)	0.883 (0.820-0.937)	0.938 (0.875-0.986)	0.809 (0.692-0.917)	0.902 (0.844-0.950)	0.540
CH Combined	0.954 (0.916-0.992)	0.883 (0.820-0.937)	0.906 (0.831-0.967)	0.851 (0.750-0.950)	0.899 (0.845-0.948)
External test set						
Crad-score	0.857 (0.770-0.944)	0.716 (0.614-0.807)	0.750 (0.586-0.905)	0.700 (0.574-0.815)	0.627 (0.469-0.753)	0.023
Hrad-score	0.893 (0.815-0.971)	0.659 (0.557-0.761)	0.929 (0.821-1.000)	0.533 (0.411-0.652)	0.634 (0.500-0.750)	0.100
Clinical	0.906 (0.847-0.965)	0.830 (0.750-0.898)	0.857 (0.704-0.967)	0.817 (0.712-0.904)	0.762 (0.625-0.862)	0.012
CC Combined	0.933 (0.883-0.983)	0.739 (0.636-0.830)	0.964 (0.870-1.000)	0.633 (0.500-0.759)	0.701 (0.567-0.810)	0.240
CH Combined	0.954 (0.914-0.994)	0.841 (0.761-0.909)	0.964 (0.885-1.000)	0.783 (0.672-0.879)	0.794 (0.667-0.886)

Crad-score, classical radiomics model; Hrad-score, habitat radiomics model; Clinical, Clinical model; CC Combined, combined clinical-classical radiomics model; CH Combined, combined clinical-habitat radiomics model.

AUC, area under the curve; ACC, Accuracy; SEN, Sensitivity; SPE, Specificity; F1, F1-Score; NPV, Negative Predictive Value; PPV, Positive Predictive Value; p, P-value obtained from the DeLong test comparing other models with the CH Combined model.

### 3.4 Performance of models in the PI-RADS 3–4 scores subgroup

The training set consisted of 173 patients who were identified as having PI-RADS score of 3–4, including 110 cases of non-csPCa and 63 cases of csPCa. There were 50 patients in the internal test set, with 33 non-csPCa and 17 csPCa in the internal test set. The external test set included 56 patients, among whom 47 were non-csPCa, and 9 were csPCa. **Table 3** and **Figure 5** present the diagnostic performance of the four models in classifying csPCa among cases with PIRADS 3–4 scores. The CH combined model integrating habitat radiomics and clinical characteristics yielded AUCs of 0.933 (0.899-0.968) in the training set, 0.869 (0.762-0.958) in the internal test set, and 0.962 (0.895-1.029) in the external test set, which was higher than that of the other models. The CH habitat model achieved higher AUCs in both the internal and external test cohorts (0.869 vs 0.865, 0.962 vs 0.908). However, the CC Habitat model exhibited superior AUC values within the training cohort.

**Table 3:** Comparison of diagnostic efficacy between four models in PI-RADS 3–4 score lesions.

Model	AUC (95% CI)	ACC (95% CI)	SEN (95% CI)	SPE (95% CI)	F1 (95% CI)	p
Training set						
Crad-score	0.928 (0.891-0.964)	0.844 (0.786-0.896)	0.651 (0.527-0.766)	0.955 (0.910-0.991)	0.752 (0.650-0.836)	0.770
Hrad-score	0.898 (0.851-0.945)	0.827 (0.769-0.879)	0.714 (0.588-0.823)	0.891 (0.824-0.944)	0.750 (0.643-0.827)	0.450
Clinical	0.862 (0.803-0.922)	0.827 (0.769-0.879)	0.667 (0.548-0.780)	0.918 (0.870-0.963)	0.737 (0.638-0.817)	0.002
CC Combined	0.95 (0.921-0.979)	0.890 (0.844-0.936)	0.841 (0.746-0.930)	0.918 (0.865-0.964)	0.848 (0.773-0.909)	0.200
CH Combined	0.933 (0.899-0.968)	0.873 (0.821-0.919)	0.730 (0.621-0.831)	0.955 (0.914-0.990)	0.807 (0.723-0.876)	
Internal test set						
Crad-score	0.772 (0.634-0.897)	0.720 (0.580-0.840)	0.471 (0.235-0.700)	0.848 (0.714-0.967)	0.533 (0.286-0.722)	0.16
Hrad-score	0.830 (0.694-0.942)	0.740 (0.600-0.860)	0.706 (0.500-0.917)	0.758 (0.607-0.900)	0.649 (0.454-0.812)	0.32
Clinical	0.784 (0.647-0.897)	0.720 (0.580-0.840)	0.647 (0.412-0.875)	0.758 (0.600-0.900)	0.611 (0.400-0.791)	0.07
CC Combined	0.865 (0.758-0.962)	0.800 (0.680-0.900)	0.765 (0.545-0.944)	0.818 (0.679-0.943)	0.722 (0.538-0.870)	0.89
CH Combined	0.869 (0.762-0.958)	0.780 (0.660-0.880)	0.647 (0.412-0.875)	0.848 (0.730-0.968)	0.667 (0.467-0.826)	
External test set						
Crad-score	0.844 (0.732-0.955)	0.732 (0.607-0.839)	0.667 (0.333-1.000)	0.745 (0.622-0.865)	0.444 (0.190-0.648)	0.38
Hrad-score	0.891 (0.777-1.005)	0.607 (0.482-0.732)	0.889 (0.625-1.000)	0.553 (0.413-0.689)	0.421 (0.210-0.600)	0.047
Clinical	0.92 (0.839-1.000)	0.893 (0.804-0.964)	0.556 (0.200-0.889)	0.957 (0.898-1.000)	0.625 (nan-nan)	0.26
CC Combined	0.908 (0.818-0.998)	0.732 (0.607-0.840)	0.889 (0.667-1.000)	0.702 (0.565-0.833)	0.516 (0.261-0.710)	0.25
CH Combined	0.962 (0.895-1.029)	0.911 (0.821-0.982)	0.889 (0.625-1.000)	0.915 (0.822-0.980)	0.762 (0.500-0.947)	

* Crad-score classical radiomics model, Hrad-score habitat radiomics model, Clinical, Clinical model, CC Combined, combined clinical features and classical radiomics model, CH Combined, combined clinical features and habitat radiomics model.

AUC Area under the curve, ACC Accuracy, SEN Sensitivity, SPE Specificity, F1 F1-Score, *p* P-value is the Delong test result of comparing different models with the CH Combined model.

**Figure 5:**
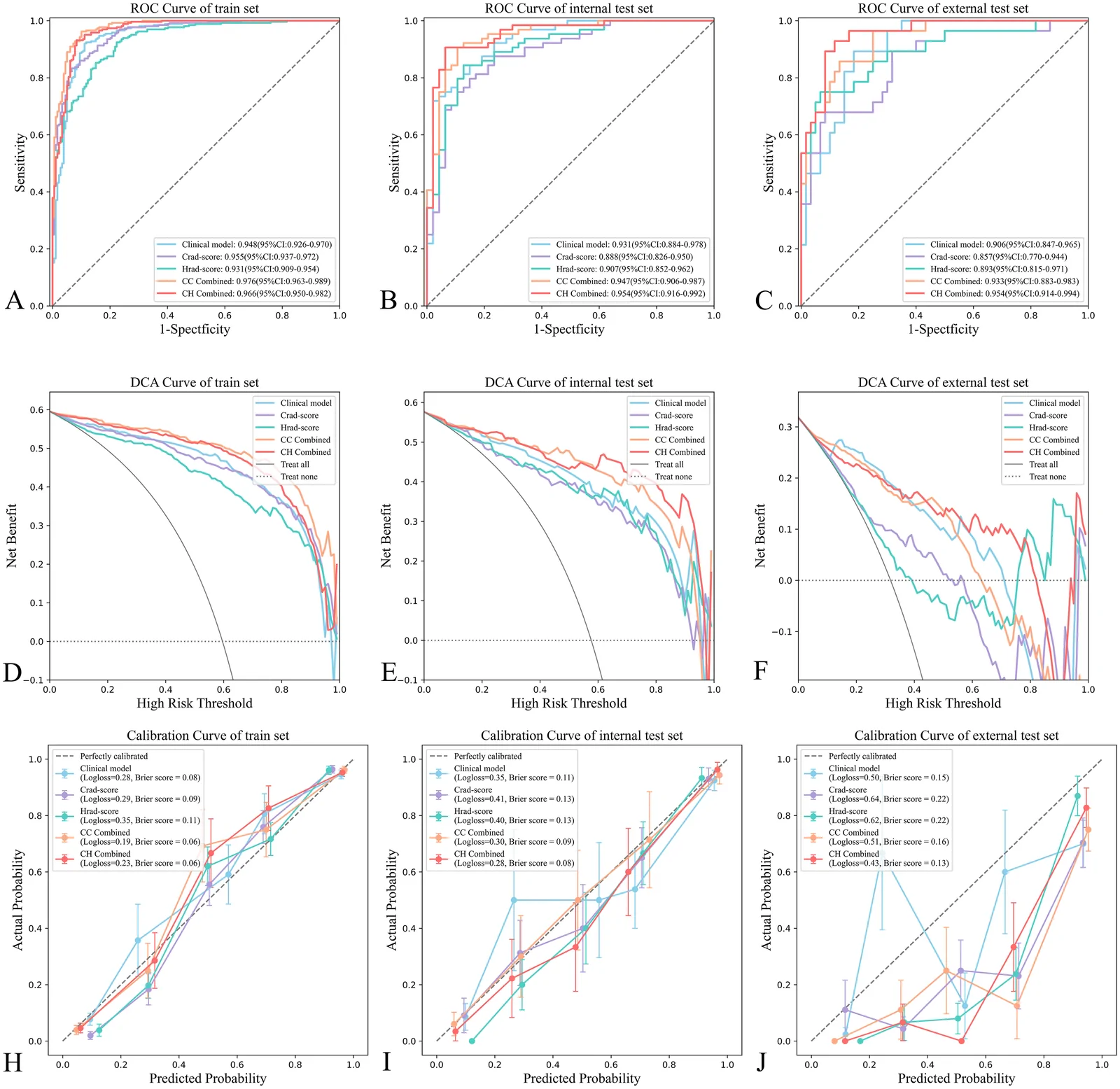
The efficacy of each model in identifying the csPCa with PI-RADS 3–4 leisions. Model evaluation; ROC curves of the different model in the train **(A)**, internal test **(B)** and external test **(C)**; the DCA curve for the train **(D)**, internal test **(E)** and external test **(F)** sets; calibration curves in the train **(G)**, internal test **(H)** and external test **(I)** sets.

## 4 Discussion

This study applied habitat radiomics analysis based on mpMRI to predict csPCa, and the CH combined model that integrated habitat radiomics with clinical features demonstrated higher diagnostic accuracy compared to the single rad-score model and clinical model, yielding AUCs of 0.966, 0.954, and 0.954 in the training, internal test, and external test sets, respectively. In PI-RADS 3–4 subgroups, the CH combined model continues to exhibit the relatively highest predictive performance in three sets, with AUCs of 0.933, 0.869 and 0.962. These findings suggest that the integrated framework of habitat analysis and clinical variables represents a promising non-invasive diagnostic paradigm for detecting csPCa. While the current results are exploratory, they demonstrate potential clinical utility and pave the way for future multicenter studies to validate the model’s incremental benefits.

Previous studies have successfully employed radiomics models to predict diagnosis efficacy in patients with PCa (17, 18). Min et al. (19) evaluate the performance of mpMRI-based radiomics signature for discriminating csPCa, achieving AUC values of 0.872 and 0.823 in the training and internal test cohorts, respectively. Li et al. (20) developed the Rad-score based on mpMRI to predict PCa, achieving AUCs of 0.979 and 0.831 in the training and external test sets. In the present study, the classical radiomics model showed good performance for the prediction of csPCa, with AUCs of 0.955,0.888 and 0.857 in the three sets, which is consistent with the findings of these previous studies.

The Hrad-score yielded higher AUCs compared to the Crad-score model in terms of prediction performance and clinical usefulness, although the difference did not reach statistical significance. Meanwhile, the Hrad-score improved the prediction of the CH combined model, which was consistent with the conclusions of prior research. The findings of Ye et al. (21) demonstrated that the predictive model developed using mpMRI habitat analysis exhibited superior performance in predicting extraprostatic extension in PCa compared to a conventional radiomics model. The analysis approach based on the entire tumor was easily impacted by the scan-related environment, but the habitat-based quantification groups comparable voxels and is therefore more robust in this instance. This could be one explanation why the habitat-based method performed better in our investigation. Another reason could be that habitat analysis, which is based on voxel signal intensity distributions, assessed subtle differences in tumor microenvironments, such as hypoxia, acidosis, immune responses, growth factors, and glucose consumption, which already occurred in phenotypic adaptation in csPCa lesion subregions.

Given the suboptimal positive detection rate of csPCa among patients with PI-RADS 3–4 score (22, 23), there is an urgent requirement to enable precise diagnosis of csPCa, which will help reduce unnecessary prostate biopsies. Our study reveals that an integrative habitat radiomics framework can stratify suspicious PI-RADS 3–4 lesions, achieving high diagnostic accuracy (AUC: 0.869–0.962). This might be attributed to intratumoral heterogeneity, which offers additional details regarding the lesion. Theoretically, in the training set, using CH combined model for biopsy decision-making, it could prevent 95.5% (105/110) unnecessary biopsies, at the cost of only a 27.0% (17/63) rate of csPCa missed diagnosis. Compared with the clinical model, the CH combined model yielded an AUC of 0.962 in the external test set; a sensitivity of 0.889 and specificity of 0.915, indicating good migration performance. From a clinical decision-support perspective, the CH combined habitat model demonstrates better potential for optimizing biopsy triage within the PI-RADS 3-4 gray zone.’

However, this study has certain limitations. First, the non**-**significant group in the external test set was limited to benign prostatic hyperplasia and lacked non**-**significant prostate cancer (non**-**csPCa) cases, which may not fully represent the spectrum of non**-**csPCa. Although this likely did not improve model performance, it may have led to an overestimation of its diagnostic effectiveness. Further studies with larger multicenter datasets are warranted to validate the model**’**s generalizability and enhance its clinical applicability. Second, the habitat subregions generated by clustering have not been pathologically validated, and their biological significance warrants further investigation. Third, the radiomics analysis relied on manual lesion segmentation, which may introduce subjectivity and inter**-**operator variability. Future work will explore automatic segmentation techniques to improve objectivity and efficiency. Fourth, PI**-**RADS scoring was performed by a single senior radiologist, and no inter**-**reader variability assessment was conducted. Although this radiologist was highly experienced, the lack of multi**-**reader evaluation may not capture the inherent variability in PI**-**RADS 3**-**4 lesion interpretation across readers. Future studies incorporating multiple readers are needed to assess the model’s robustness and generalizability across different scoring practices.

## 5 Conclusion

Overall, the CH combined model demonstrated efficacy in predicting csPCa and is expected to enhance clinical grading assessments. This habitat analysis-based nomogram may serve as a noninvasive tool to provide additional diagnostic value for risk stratification, particularly in patients with PI-RADS 3-4 lesions, where clinical decision-making remains challenging.

## Data Availability

The original contributions presented in this study are included in the article and **Supplementary Materials**. Due to hospital data privacy regulations and the retrospective nature of this clinical study, the raw patient imaging and clinical data are not publicly available. Further inquiries to access the relevant datasets can be directed to the corresponding author upon reasonable request.
