# Supplementary material for: MRI-based habitat radiomics analysis for predicting clinically significant prostate cancer: a retrospective dual-center study
Source: Front Oncol. 2026 Sep 21;16:1831344. doi: 10.3389/fonc.2026.1831344 (PMC13634948; doi:10.3389/fonc.2026.1831344)
Supplement: Supplementary file 1 [file Table1.docx]

**Table 1** MRI acquisition parameters for Center 1

|  | Sequence | Thickness（mm） | Spacing（mm） | Repetition time（ms） | echo time（ms） |
| --- | --- | --- | --- | --- | --- |
| GE Discovery 3.0T MR | T1WI | 3.0 | 0.5 | 620 | 7.8 |
|  | T2WI |  |  | 7973 | 122 |
|  | DWI |  |  | 2600 | 68.2 |
| GE Signa HDxt 3.0T MR | T1WI | 6.0 | 1.5 | 550 | 7.0 |
|  | T2WI |  |  | 2760 | 68 |
|  | DWI |  |  | 4150 | 76.4 |
| GE Signa HDxt 1.5T MR | T1WI | 5.0 | 1.5 | 629 | 10.5 |
|  | T2WI |  |  | 3440 | 102 |
|  | DWI |  |  | 4450 | 68.2 |

**Table 2** MRI acquisition parameters for Center 2

|  | T1WI | T2WI | DWI |
| --- | --- | --- | --- |
| TR（ms） | 500 | 3800 | 4700 |
| TE（ms） | 11 | 78 | 83 |
| Thickness（mm） | 3.5 | 3.5 | 3.5 |
| Spacing（mm） | 1 | 1 | 1 |
| FOV（mm） | 22 | 22 | 22 |
| Number of samples | 3 | 3 | 3 |

* The DWI parameter is an image parameter with a B value of 3000 s/mm^2^.
